# DIGNITY: Feedback From Rural Nursing Home Stakeholders via Community Engagement Studios

**DOI:** 10.1093/geroni/igaf122.2069

**Published:** 2025-12-31

**Authors:** Susan Ryan, Liza Behrens, Kimberly Van Haitsma, Jennifer Kraschnewski, Marie Boltz

**Affiliations:** Center for Innovation, Linthicum, Maryland, United States; The Pennsylvania State University, University Park, Pennsylvania, United States; The Pennsylvania State University, University Park, Pennsylvania, United States; Penn State College of Medicine, Hershey, Pennsylvania, United States; The Pennsylvania State University, University Park, Pennsylvania, United States

## Abstract

Prior intervention development work identified the need to involve rural nursing home (NH) stakeholders in the initial development of DIGNITY. To meet this need, Community Engagement (CE) Studios were held to obtain feedback on study design and procedures (intervention protocol, program materials, implementation and evaluation plan). Four rounds of CE Studios were conducted with self-identified NH leaders, staff, regulators, and resident advocates (n = 2), and rural NH community staff, residents with dementia, and family members (n = 2). All CE studios were held in person and followed institutional guidelines. Each CE studio followed a similar format including: a welcome and ice breaker exercise, an introduction to the DIGNITY program, a risky preference card sort activity, and a detailed review of relevant DIGNITY program manual procedures and tools. Audio recordings and written field notes recorded participant responses. Rapid content analysis provided themes for reporting results in a standardized feedback loop. In total, 39 Community Experts participated in CE studios between July 2022 and August 2024. The Experts represented NH leadership (n = 3), nursing staff (n = 10), social work (n = 1), activities staff (n = 1), residents with dementia (n = 4), family members (n = 4), surveyors (n = 15), and a federal regulator (n = 1). Just under half of the participants were working/living in a rural NH community (n = 16). Experts primarily suggested changes to program materials and procedures, including the DIGNITY Practice Model, Personal Risk Portfolio, Risk and Impact Worksheet, and Decision-Making Capacity Assessment Tool. Experts were also concerned about program costs. Future work needs to implement all expert suggestions into the intervention manual.

